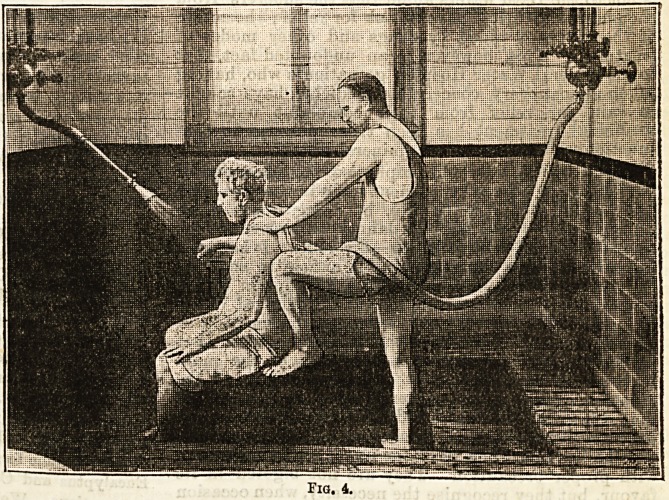# Treatment of Rheumatoid Arthritis

**Published:** 1892-10-08

**Authors:** 


### BATH ROYAL MINERAL WATER HOSPITAL.

### Treatment of Rheumatoid Arthritis.

On Saturday, May 21st, 1892, with the usual signs of rejoicing, the Bath Royal Mineral Water Hospital

Fig. 1.

celebrated its third jubilee. Few of our provincial hospitals can claim a record extending over a century and a half, a record of 150 years of useful work, and of benefit to the afflicted among mankind. It is on occasions like this, occasions that mark the lapse of time, and that stand out as mile-stones on the road of centuries, that it is well for us to pause and to review the work that is being done at institutions such as this, in order that we may assure ourselves that the trust, which has been bequeathed to us by our forefathers, is being faithfully administered.

It is with this object in view that we ask the readers' attention to the following short account of one branch, among many others, of the good work that is being done at the Royal Mineral Water Hospital, namely, with regard to the treatment carried out there of rheumatoid arthritis. This mysterious disease, as seen at the Bath Hospital, varies very much in its intensity. First there are those cases of only a few months' standing, in which the symptoms are very slight, and the joint affections quite insignificant; then there is a much larger class, where the disease is of a pronounced type, and this class includes by far the larger number of the rheumatoid patients; and, finally, there is a last group in which the first onset dates back fifteen, or it may be twenty, years, and where its ravages during the best part of a lifetime have left their marks in a condition of hopeless crippling and disorganisation of the joints. For the first two groups much may be done by a proper line of treatment, and in those cases that have not gone too far the disease may be altogether arrested; but for the third group, what can be done? Truly, little or nothing; their pains may be to a certain extent alleviated by the warm bathing, and their general health improved by the change of air, the regular hours, and the good diet of the hospital; but no amount of washings can ever restore a joint in which the cartilage has been eroded, and the bones eburnated, or in which the articulating surfaces have become ankylosed together into a permanent condition of synostosis. Yet many patients in this desperate

Fig. 2.

state go to the Mineral Water Hospital under the impression that they will be cured, only, however, to suffer disappointment when the disillusion comes. For such as these it is necessary to remark that the days of miracles are past; that the streams of Bethesda, if they still retain their ancient virtue, do not come to the surface among the springs of Bath, and that the cure which they seek can only be found in the oblivion of the waters of Lethe.

It is, of course, impossible in an article like the present to give any description of rheumatoid arthritis, or to enter into a discussion as to its nature, nor is it our desire to do so. Much that is good, bad, and indifferent has been written about it; and for fuller information with regard to it the reader is referred elsewhere. Let it suffice to draw attention to the accompanying print, a reproduction of a photograph, which will serve to remind our readers of the general features of the disease (Fig. 1). The wasted muscles, the enlargements of the joints, especially of the wrists and phalanges, due to synovial thickening, and the facial aspect of the patient, which in itself is of considerable diagnostic value, are all seen here, and, in their silence, they speak more eloquently of the patient's condition than could any words of ours.

We come then to a description of the treatment of this disease as carried out at the Bath Hospital. For this purpose it will be convenient to divide the subject into two headings; in the first place to describe the thermal, and in the second place the dietetic and therapeutic treatment.

Fig. 3.

First then as to the thermal treatment. The mineral waters of Bath, which rise at a temperature of about 115 deg. to 120 deg., are found to be of the greatest service in rheumatoid arthritis if used in the proper cases and in a manner suitable to the condition of the patient. They are employed in two ways, firstly for drinking, and secondly for bathing. As a rule the patient drinks twenty ounces in the course of the day, half in the morning before breakfast, and half the last thing at night; but the amount varies with the individual case, and some are given as much as thirty ounces in the twenty-four hours. Taken internally, the waters act by stimulating the digestive organs to an increased vigour, by increasing the normal secretion of the liver, the kidneys, and the skin, and finally, through their tonic action, by promoting the general nutrition of the system.

Next as to the bathing. The ordinary bath, with which the patient generally commences, has a capacity of 3,682 gallons, and in it the water is of a depth of 4 ft. 6 in. (Fig. 2). The temperature is varied, for the different class of cases, from 98 deg. to 104 deg. While bathing, the patient is encouraged to work the affected joint as much as possible, and also to apply friction to the part, either with the hand, or what is better, with the flesh brush, while at intervals he makes use of the "douche," directing the stream of water on to the crippled limb. Other things being equal, he remains in the bath for a quarter of an hour, and bathes on alternate mornings. On leaving the bath he goes to his bed, and there lies, packed in blankets, while he recovers his equilibrium. The word "douche" has just been made use of and needs a little explanation. In the technicality of the thermal treatment there are two forms of "douches," the wet and the dry "douche." At first sight it would seem that both are wet, for both consist of a stream of water proceeding from a hose pipe; but the difference is this, that the "wet douche" is directed on to the desired part while the patient is bathing, and therefore under water, whilst the "dry douche" is applied locally when he is not bathing. In using the wet douche while the temperature of the bath is probably 98 deg. to 100 deg., that of the douche itself, which falls on the joint at a considerable pressure, is about 105 deg. to 110 deg. These douches are found to be of great value, and seem to act as local baths, producing hyperæmia of the skin, and thus promoting absorption and reducing swellings.

Next to come to the massage baths, which have been in use at the hospital about three years, and whose efficacy is now thoroughly established. They are built on the model of the baths at Aix, and may be described as a combined process of dry douching and massage, which, translated into the vulgar tongue of the patients, is called "deucing and mashing" (Fig. 3). The accompanying photograph shows a patient in the hands of the masseur; he is seated on a chair, and upon his back and shoulders there is directed, from a rose pipe, a many-streamed current of the hot mineral water, whilst, at the same time, the operator, with his "dry douche" of the same health-producing element, plays upon his crippled limb, and kneads it the while with a skill that comes only of long experience (Fig. 4). These baths are of special value in cases of rheumatoid arthritis. Under their influence the enlarged joints tend to decrease, and the wasted muscles to recover their tone, while the mobility of the limb is aided; and, finally, what is quite as important as all these, the general health of

Fig. 4.

the patient is improved by an increased appetite and a healthy action of the skin.

Other baths, and many minor details of the thermal treatment of rheumatoid arthritis, remain untold, but space presses, and we pass on to a consideration of the dietetic and therapeutic treatment of this disease.

Rheumatoid arthritis is essentially a debilitating disease, or, rather, it might be said more truly to be a disease of debility. It is necessary, therefore, in all cases to improve, as far as may be, the patient's general condition. With this object in view a liberal diet must be maintained. The old practice, depending for its existence upon the erroneous notions as to the nature of the disease, and the false assumption that it was closely allied to gout, of restricting the diet and avoiding stimulants is fast dying out. At the Mineral Water Hospital the necessity of this point of good feeding is luckily fully recognised. There is a tendency, however, in this disease to digestive troubles, when strong food is not well borne, and this forms a grave obstacle in the maintenance of the patient's strength. In these cases the sheet anchor is milk, supplemented with beef tea, eggs, &c., and light stimulants. Next as to drugs. That which is above all others important is cod-liver oil; and, indeed, it had better have been included under the dietary, for it is more of the nature of a food than a medicine; beginning gently with teaspoonful doses, the average rheumatoid patient soon works up to being able to take a tablespoonful twice a day with the greatest benefit. Medicines, such as the iodide of potassium and the tincture of iodine, are of great service, and for the rest iron, arsenic, and strychnine are given as the case requires.

For the pyrexial attacks, which are by no means uncommon during the progress of a case of rheumatoid arthritis, salicylates have a marked effect, both in reducing the temperature and allaying the pain in the joints. The tendency to night sweats, from which the patient often suffers, is combated with sulphonal, which seems to act especially upon this condition, quite apart from its virtue as a sleep-producing agent.

It will be seen by the above list of medicines that it is not to the Bath waters alone that the sufferers from rheumatoid arthritis have to look for relief; the action of the waters is aided by drugs and other modes of treatment. This, however, is an undoubted fact, that to the Mineral Water Hospital go patients who, having taken almost every conceivable medicine, have sought in vain for relief from their complaint until the medicinal treatment has been there pursued in conjunction with the use of the mineral waters.

In conclusion, we would emphasise the necessity of beginning the thermal treatment early in the case; and not waiting until it is too late, by doing which much disappointment, and perhaps danger, may occur to the patient, and to the Bath waters—discredit. It has been impossible within the scope of this article to enter as fully as we should have liked into a description of the excellent work that is being done at the Mineral Water Hospital. We chose for our theme the treatment there carried out for rheumatoid arthritis, and we trust that we have given our readers a fair idea of the main features of that treatment. The upholders of the curative virtues of the mineral water do not profess that it is an all-healing remedy, but they maintain, and they are justified in their thesis, that it is of the greatest possible service when used for appropriate cases, and with proper precaution. They are not bigoted in its favour, but they recognise the necessity, when occasion occurs, of supplementing its action from the storehouse of the pharmacopœia. It is the wisdom of the physician that teaches him, in order that he may discomfit his foe, at what point he must rally his forces, and when to bring forth certain weapons from his armoury. Finally, we would wish prosperity, and a further range of usefulness to the Royal Mineral Water Hospital, and to Bath, "the Queen of the West"—the "City of the Waters of the Sun."

## Figures and Tables

**Fig. 1. f1:**
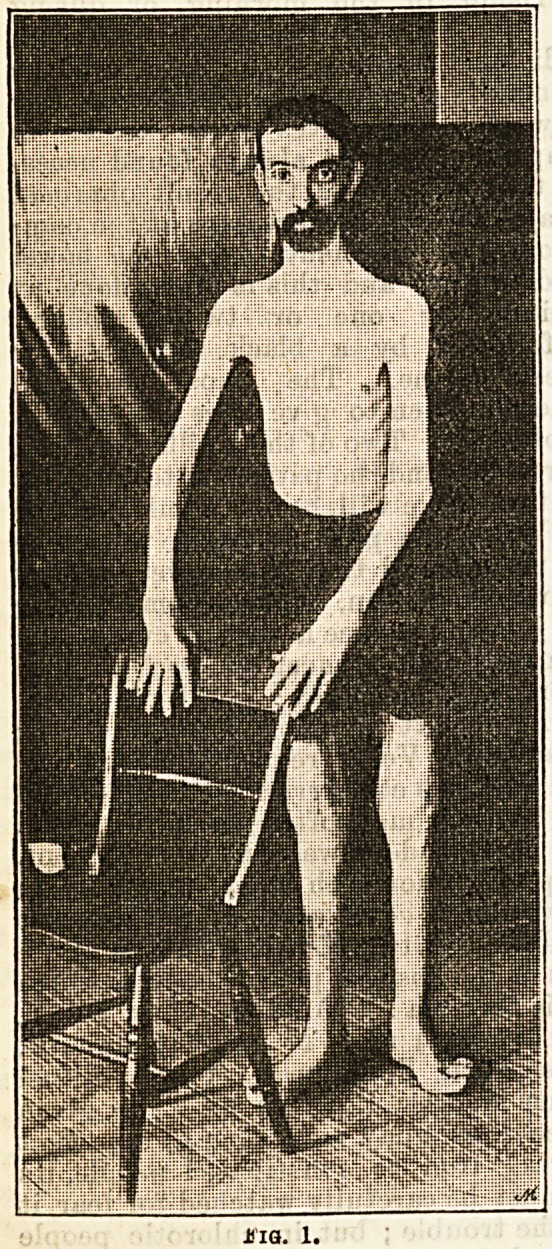


**Fig. 2. f2:**
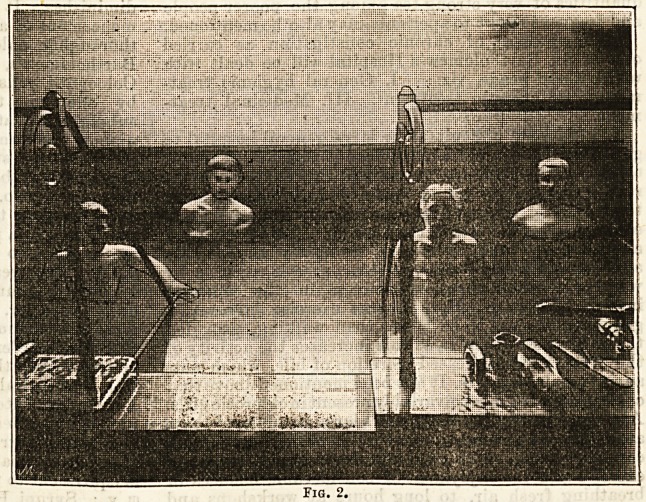


**Fig. 3. f3:**
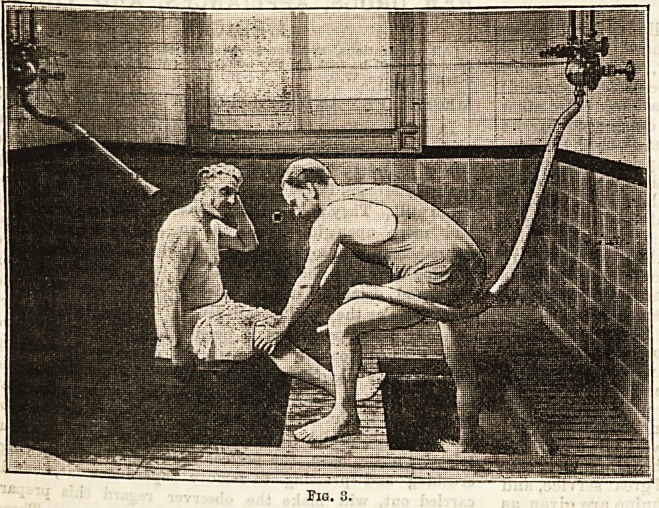


**Fig. 4. f4:**